# Motor Sequence Learning Deficits in Idiopathic Parkinson’s Disease Are Associated With Increased Substantia Nigra Activity

**DOI:** 10.3389/fnagi.2021.685168

**Published:** 2021-06-14

**Authors:** Elinor Tzvi, Richard Bey, Matthias Nitschke, Norbert Brüggemann, Joseph Classen, Thomas F. Münte, Ulrike M. Krämer, Jost-Julian Rumpf

**Affiliations:** ^1^Department of Neurology, University of Leipzig, Leipzig, Germany; ^2^Department of Neurology, University of Lübeck, Lübeck, Germany; ^3^Institute of Neurogenetics, University of Lübeck, Lübeck, Germany; ^4^Department of Psychology, University of Lübeck, Lübeck, Germany; ^5^Center of Brain, Behavior and Metabolism, University of Lübeck, Lübeck, Germany

**Keywords:** Parkinson’s disease, motor sequence learning, fMRI, dynamic causal modeling, substantia nigra, hippocampus

## Abstract

Previous studies have shown that persons with Parkinson’s disease (pwPD) share specific deficits in learning new sequential movements, but the neural substrates of this impairment remain unclear. In addition, the degree to which striatal dopaminergic denervation in PD affects the cortico-striato-thalamo-cerebellar motor learning network remains unknown. We aimed to answer these questions using fMRI in 16 pwPD and 16 healthy age-matched control subjects while they performed an implicit motor sequence learning task. While learning was absent in both pwPD and controls assessed with reaction time differences between sequential and random trials, larger error-rates during the latter suggest that at least some of the complex sequence was encoded. Moreover, we found that while healthy controls could improve general task performance indexed by decreased reaction times across both sequence and random blocks, pwPD could not, suggesting disease-specific deficits in learning of stimulus-response associations. Using fMRI, we found that this effect in pwPD was correlated with decreased activity in the hippocampus over time. Importantly, activity in the substantia nigra (SN) and adjacent bilateral midbrain was specifically increased during sequence learning in pwPD compared to healthy controls, and significantly correlated with sequence-specific learning deficits. As increased SN activity was also associated (on trend) with higher doses of dopaminergic medication as well as disease duration, the results suggest that learning deficits in PD are associated with disease progression, indexing an increased drive to recruit dopaminergic neurons in the SN, however, unsuccessfully. Finally, there were no differences between pwPD and controls in task modulation of the cortico-striato-thalamo-cerebellar network. However, a restricted nigral-striatal model showed that negative modulation of SN to putamen connection was larger in pwPD compared to controls during random trials, while no differences between the groups were found during sequence learning. We speculate that learning-specific SN recruitment leads to a relative increase in SN- > putamen connectivity, which returns to a pathological reduced state when no learning takes place.

## Introduction

The clinical diagnosis of Parkinson’s disease (PD), the second most prevalent neurodegenerative disorder worldwide, is based on the cardinal motor symptoms bradykinesia, muscle rigidity, and tremor (Berardelli et al., 2013). These PD-defining motor symptoms are mainly related to the degeneration of dopaminergic neurons in the substantia nigra (SN) pars compacta (Fearnley and Lees, 1991) and loss of neurons in the nigro-striatal pathway projecting primarily to the putamen (Dauer and Przedborski, 2003). However, PD also affects several other neurotransmitter systems, including cholinergic, noradrenergic, glutaminergic, and GABAergic pathways (Calabresi et al., 2006; Francis and Perry, 2007; Brichta et al., 2013). Persons with PD (pwPD) often also suffer from cognitive dysfunctions that compromise working memory, executive functions and attention (Williams-Gray et al., 2007) as well as implicit motor learning and retention of motor skills (Redgrave et al., 2010). Early procedural learning studies showed that pwPD have specific learning-related deficits (Pascual-Leone et al., 1993; Jackson et al., 1995; Doyon et al., 1997). These deficits were already evident in very early stages of the disease and even detectable when the asymptomatic hand was tested in unilateral de novo pwPD (Dan et al., 2015). However, these findings were not always consistent (Smith et al., 2001; Werheid et al., 2003; Seidler et al., 2007), probably due to strong variability in disease progression (Muslimović et al., 2007; Stephan et al., 2011) and pharmacotherapy (Kwak et al., 2012, 2010) as well as variability in task demands. A meta-analysis revealed that while pwPD have clear deficits in their ability to implicitly learn a motor sequence compared to healthy controls, they were still able to improve their performance with practice, albeit to a lesser degree (Hayes et al., 2015).

To investigate the underlying neural mechanisms of motor sequence learning (MSL) deficits in PD, previous research has employed imaging methods such as positron emission tomography and functional magnetic resonance imaging (fMRI). Early studies showed that acquisition and retention of a motor sequence recruits different brain regions in PD compared to controls (Nakamura et al., 2000; Mentis et al., 2003) and leads to over-activation of premotor and parietal areas as well as the cerebellum in PD (Sabatini et al., 2000; Wu and Hallett, 2005; Caproni et al., 2013). Effective connectivity analysis showed that interactions between cerebellar and premotor areas were significantly reduced in PD during the automatic phase of MSL (Wu et al., 2010). Thus, evidence suggests that increased activity and decreased connectivity within the motor network may underlie MSL deficits in PD.

In healthy subjects, learning a motor sequence activates a distributed network including striatum, thalamus as well as motor cortical areas, parietal cortex, dorsolateral prefrontal cortex and cerebellum (meta-analysis by Hardwick et al., 2013). Theoretical models suggest that motor learning is implemented through specific cortico-striatal and cortico-cerebellar circuits, which mediate different learning stages (Doyon et al., 2009). Previously, we tested this model in healthy subjects and found that learning negatively modulated connections from M1 to cerebellum (Tzvi et al., 2014) but also between putamen and cerebellum (Tzvi et al., 2015, 2017). These results suggest that besides the cerebellum, the putamen may also play an important role in acquisition of new motor sequences.

In this study, we aimed to investigate how the dysfunctional nigro-striatal dopaminergic system in PD affects neural activity and connectivity in a motor learning network, while pwPD perform an implicit MSL task, concurrent to fMRI. We hypothesized that dysfunction of the nigro-striatal system in PD will affect activity and connectivity in the cortico-striato-thalamo-cerebellar network. Specifically, we expected that learning deficits in pwPD would be associated with neural changes in the striatum, and lead to altered network interactions underlying MSL.

## Materials and Methods

### Participants

Twenty-three persons with Parkinson’s disease (see Table 1 for demographics) volunteered to participate in the study. Idiopathic PD was diagnosed by expert neurologists (M.N. and N.B.) and the severity of motor symptoms was assessed according to the Unified Parkinson’s disease Rating Scale part III (UPDRS III; Fahn et al., 1987). PwPD were recruited from the outpatient clinic of the Department of Neurology, Lübeck University Hospital. Upon recruitment, pwPD were first tested for their general cognitive abilities using the Mini-Mental State Examination (Pangman et al., 2000). Those who scored more than 20 out of 30 points on the Mini-Mental State Examination and less than 40 points on UPDRS III were eligible to participate. Note that this includes pwPD that may suffer from light dementia which should not affect implicit motor sequence learning (Hong et al., 2020). In addition, we used a short pre-task test block to ensure that finger-tapping skills required to perform the task were intact. From this cohort, we had to exclude five pwPD due to diagnosis of depression, or severe cortical atrophy detected in the anatomical scan (see Table 1 for more details). Two additional pwPD were rejected from analysis of task-fMRI data due to bad performance (one fell asleep during the task and the other made more than 50% errors). Thus, we included sixteen pwPD (10 males; 6 females; age: 46–77; mean age: 64.3). To this sample we age-matched 16 neurologically healthy controls (7 males; 9 females; age: 53–77; mean age: 62.6) who were recruited from the general community. All participants were right-handed (except for one pwPD) and had normal or corrected to normal vision. Participants gave informed written consent prior to study participation. The study was approved by the Ethics Committee of the University of Lübeck.

**TABLE 1:** Characteristics of persons with PD.

ID	Age	Gender	MMSE	UPDRSIII	DD	LED
*P01	48	m	30	20	7	760
P02	65	f	22	10	−	325
P03	74	m	24	22	13	1,169
*P05	53	f	30	16	8	−
P06	46	m	29	10	7	565
P07	70	f	29	21	9	250
P09	65	m	30	34	3	179
P10	54	m	25	34	3	905
**P11	66	m	25	32	5	782
P12	61	m	28	36	5	814
**P13	48	m	27	25	5	1246
P15	69	m	28	39	10	450
P16	56	f	29	37	2	210
*P18	75	m	26	21	2	563
P19	60	m	29	30	1	100
P30	69	m	30	11	10	766
P33	77	f	29	11	6	430
P35	70	m	30	14	19	650
P36	61	f	29	9	3	400
P37	61	f	30	5	12	726
P38	70	m	28	8	3	320

*Participants excluded from the study. **Participants excluded from behavioral, task-based fMRI analyses and DCM analysis. –Missing data.

### Experimental Paradigm and Task Design

**FIGURE 1:**
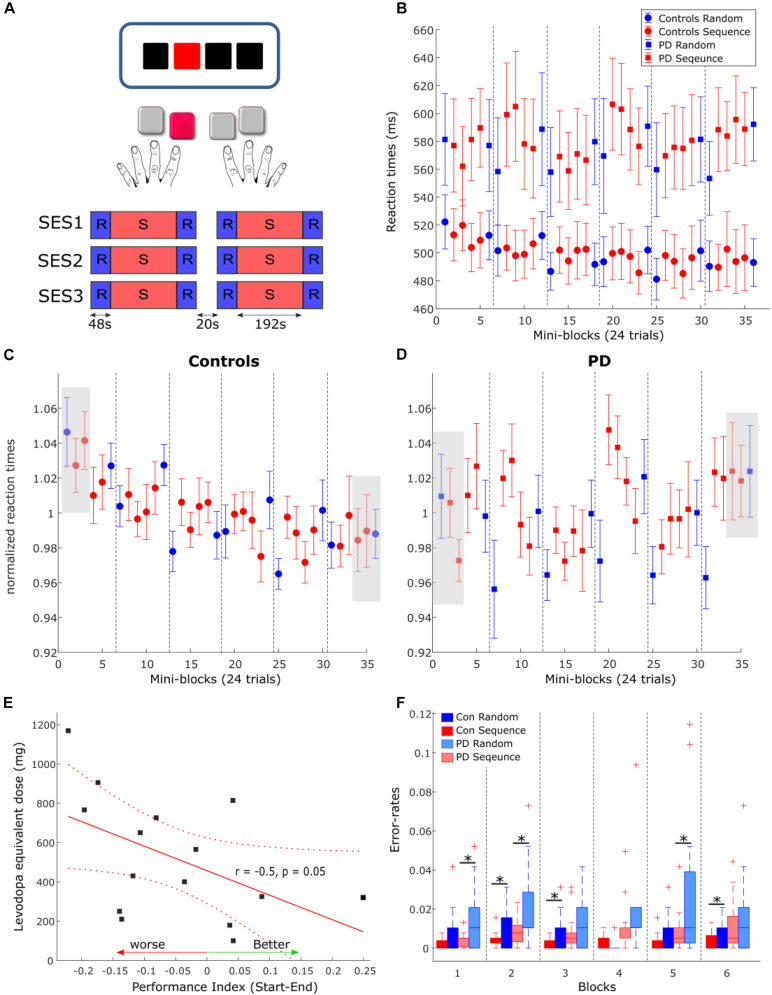
**(A)** Task and experimental design. In each trial, subjects viewed three black squares and one red square which served as the target. Subjects were instructed to press the button corresponding to the location of the red square. The task consisted of three MRI sessions (SES1-SES3) including two blocks each, amounting to a total of six blocks. 20 s breaks were introduced between sessions and blocks. **(B)** Reactions times in PD (squares) and controls (circles) in each condition and each mini-block of 24 trials. Error-bars are standard error of the mean across subjects in each group. Dashed gray lines depicts the breaks between the blocks. **(C,D)** Normalized reaction times in healthy controls **(C)** and PD **(D)**. Gray boxes show the blocks used for comparison of performance between groups. **(E)** Correlation between the performance index, calculated as the differences between the first three blocks of normalized RTs and the last three blocks, and the levodopa equivalent dose. **(F)** Error rates in each condition and each block for PD (light colors) and healthy controls. Asterisks mark significant differences within the group.

Participants performed a modified version of the serial reaction time task (Nissen and Bullemer, 1987) while lying supine in the magnetic resonance imaging (MRI) scanner after a short familiarization with the task. PwPD were under their standard dopaminergic medication (Table 1) during task performance. Visual stimuli were delivered through MR-compatible goggles worn by the subjects. In each trial, four squares were presented in a horizontal array, with each square (from left to right) associated with the following four fingers: middle finger left hand, index finger left hand, index finger right hand, middle finger right hand (Figure 1A).

Participants were instructed to respond to the red colored square with the corresponding button on an MRI-compatible keypad, one for each hand, as precisely and quickly as possible. Stimuli were presented either in a pseudorandom order (RND) or as a 12-items-sequence (SEQ, “1-2-1-4-2-3-4-1-3-2-4-3”). Participants were not aware of the existence of any patterns in the stimuli. Pseudorandom orders were generated using Matlab (Natick, MA) such that items were not repeated. The task consisted of three MRI sessions including two blocks each, amounting to a total of six blocks (Figure 1A). Each block contained eight repetitions of the 12-element sequence (i.e., 96 trials) as well as 24 random trials before and after each sequence block. A 20 sec break was introduced between the blocks during which participants were instructed to fixate on a black cross in the center of the goggle screen. Visual stimuli were presented until the onset of a button press or the onset of the next trial. The inter-stimulus interval was constant at 2,000 ms. We used Presentation^®^ software (Version 16.3^1^) to present stimuli and to synchronize the stimulus presentation and the MR functional sequences.

### Behavioral Analysis

We computed the median reaction time across 24 trials (mini-blocks) in each subject and for each of the task conditions (SEQ, RND). SEQ trials were then averaged across four mini-blocks and RND trials were averaged across the mini-block preceding and mini-block following the SEQ material. To assess the learning effects, we used mixed effects ANOVA with factors condition (SEQ, RND) and Block (blocks 1–6) as within-subject factors and Group (PD, controls) as between-subject factor.

For the error-rates, both wrong button presses and missing responses were regarded as errors. In each block and each condition, we divided the number of errors by the total number of trials in that block (SEQ: 96 trials, RND: 48 trials). Since the error-rates did not distribute normally, we used the Wilcoxon signed rank test to explore condition differences within the group, and the Wilcoxon rank-sum test to explore differences between groups (PD, controls). The false discovery rank (FDR) test was used to correct for multiple comparisons when testing for each of the six blocks separately.

To quantify performance in the task we specified two measures: the Performance Index and the Learning Index (see also Table 2). The performance index represents changes in learning of stimulus-response associations across time. It has been previously shown that stimulus-response associations are mediated by the striatum (Hiebert et al., 2014) and that pwPD on dopaminergic medication are specifically impaired in this type of learning (Vo et al., 2014; Hiebert et al., 2019). Here, the Performance Index was defined as the difference between normalized RTs, averaged across the first three blocks of the task (one random and two sequence blocks with 24 trials each) and normalized RT averaged across the three last blocks of the task (see gray squares in Figures 1C,D). The Learning Index represents learning of the hidden 12-item sequence and involves distributed brain regions such as the frontal, parietal and motor cortices as well as the striatum and cerebellum. We defined the Learning Index to be the difference in averaged RTs across blocks 3–6, between sequence and random conditions.

**TABLE 2:** Behavioral measures in the serial reaction time task.

Name	Definition
Performance index	Normalized RTs in each subject. Represents individual differences in performance across time (Figures 1C–E, 3C). Measured as the relation between RTs in each mini-block divided by the averaged RT across the entire task.
Learning index	Difference in reaction-times between sequence and random blocks (Figures 3F, 4D,E). Measured as the differences in averaged RTs across blocks 3–6, between sequence and random.

### MRI Data Acquisition and Pre-processing

The MR data were recorded using a 3T Philips Achieva head-scanner at the Institute of Neuroradiology, University of Lübeck. Functional MRI (fMRI) data (T_2_^∗^) were collected using blood oxygen level dependent (BOLD) contrast in three sessions with 300 volumes each and a gradient-echo EPI sequence following these specifications: repetition time TR = 2,000 ms, echo time TE = 30 ms, flip angle = 90°, matrix size 64 × 64, FOV = 192 × 192 mm with a whole brain coverage, ascending slice order of 3 mm thickness, and a 0.75 mm gap to avoid crosstalk between slices, in-plane resolution of 3 × 3 mm, and SENSE factor of 2. Subsequently, a high resolution T_1_-weighted structural image was acquired with FOV = 240 × 240 mm; matrix = 240 × 240; 180 sagittal slices of 1 mm thickness.

Preprocessing of fMRI data was performed using SPM12 software package^2^. The preprocessing included correction for differences in image acquisition time between slices, a six-parameter rigid body spatial transformation to correct for head motion during data acquisition, co-registration of the structural image to the mean functional image, gray and white matter segmentation, and spatial normalization of the structural image to a standard template (Montreal Neurological Institute, MNI). In order to reduce the influence of motion and unspecific physiological effects, a regression of nuisance variables from the data was performed. Nuisance variables included white matter and ventricular signals and the six motion parameters determined in the realignment procedure. Spatial normalization of the functional images was applied using the normalization parameters estimated in the previous preprocessing step and resampling to 3 × 3 × 3.75 mm. Finally, spatial smoothing with a Gaussian kernel of 8 mm full width half maximum was applied. In order to assure that signals from sub-cortical structures are not noise-contaminated and thus invalid, we also computed the temporal signal-to-noise ratio (tSNR) by averaging the mean of the time course signal intensity at each voxel (in smoothed, normalized space) and dividing it by the temporal standard deviation (Parrish et al., 2000). This procedure was done for each subject separately and then averaged across the group (PD, controls). In Table 3, we report for all activation clusters the corresponding tSNR value at the peak voxel. Note that the brain stem indeed has reduced tSNR compared to other structures but the tSNR is large enough to extract meaningful signals from this region.

**TABLE 3:** fMRI task activations (voxel level: *p* < 0.001).

Region	MNI-coordinates			*t*-value	n-voxels tSNR (PD, controls)	
**Group (PC, Cont) × SES (1,3)**						
Right premotor cortex	50	−4	54	4.33	197	146.5, 126.3
Right inferior frontal gyrus	34	8	−12	4.23	25	189.7, 178.8
Left superior temporal gyrus	−42	−24	−4	4.17	63	209.6, 204.9
Left cerebellum crus II	−32	−76	−46	4.12	100	136.2, 153.2
Left Insula	-36	12	−14	4.04	110	131.7, 119.7
Right superior temporal pole	54	12	−20	3.85	12	152.5, 149.7
Left hippocampus	−26	−16	−10	3.78	89	103.6, 113.2
**Group (PC, Cont) × Cond (SEQ, RND)**						
*Right substantia nigra	14	−18	−6	4.47	12	105.6, 105.2
*Right brainstem	8	−30	−4	4.02	205	76.1, 74.1
*Left brainstem	−6	−32	−6	3.91	162	82.4, 73.2
Left superior parietal lobule	−20	−52	70	4.07	65	156.0, 134.1
Right hippocampus	24	−22	−14	3.75	23	108.4, 108.6
Left thalamus	−12	−12	0	3.45	16	140.3, 133.8

*p = 0.05, family-wise error corrected across the cluster.

### Task-Based fMRI Statistical Analysis

Imaging data was subsequently modeled using the general linear model (GLM) in a block design. Linear regressors were obtained for each of the experimental conditions (SEQ and RND) and each session (SES1, SES2, SES3) in each subject. First level GLM analysis thus contained six experimental blocks, two for each session, modeled as a box function with the duration of each block and convolved with a hemodynamic response function. Movement related parameters from the realignment process were included in the GLM as regressors of no-interest to account for variance caused by head motion. We applied a high-pass filter (256 s) to remove low-frequency noise. First-level contrast images were generated using a one-sample *t*-test against rest periods between the blocks.

We analyzed contrast images from each participant on the second level using a random effects model. To investigate general task-related differences in activity between the groups, we performed a flexible factorial analysis accounting for main effects of Group (PD, Controls), Session (SES1, SES3), and Group × Session interaction. In addition, we explored group differences in sequence-learning activity by collapsing data across all SEQ sessions and all RND sessions and performing a flexible factorial analysis accounting for main effects of Group (PD, Controls), Condition (SEQ, RND), and interaction effects. Statistical significance was established using a whole-brain voxel-level threshold of *p* = 0.001 and cluster level of *p* < 0.05, FWE corrected over the entire cluster. To analyze interactions effects, we used the rfxplot toolbox (Gläscher, 2009) to extract the contrast estimates from significant voxels in a sphere (radius = 4 mm) around the peak activity.

We additionally calculated a metric for head motion using the translation parameters: x—left/right, y—anterior/posterior, z—superior/inferior. These parameters represent frame-wise displacement in 3D:$\sqrt{x^{2}+y^{2}+z^{2}}$. This displacement parameter was then averaged across all volumes in each session and then across sessions to produce a mean motion parameter (in mm) for each subject. We then compared the mean motion parameter between pwPD and controls using a two-sample *t*-test. One control subject was excluded from the fMRI analysis due to excessive head movements resulting in a sample of 15 healthy controls and 16 pwPD. Note that this subject was included in the behavioral analyses. After this exclusion, analysis of head motion using displacement revealed no differences (*p* > 0.1) between PD (0.69 ± 0.41) and controls (0.67 ± 0.44).

### Dynamic Causal Modeling of the Cortico-Striato-Thalamo-Cerebellar Network

**FIGURE 2:**
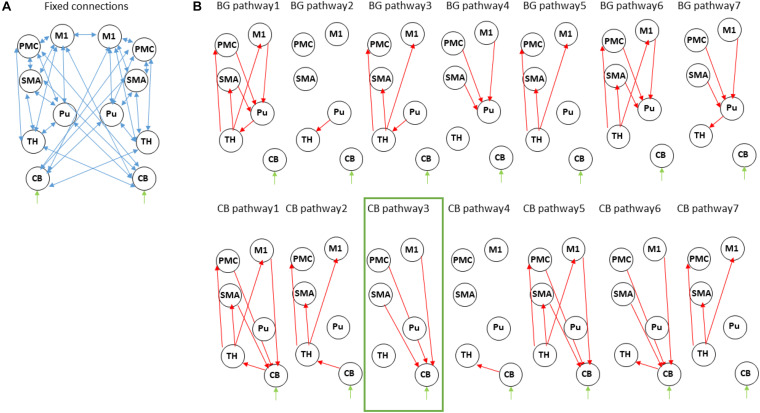
Dynamic causal modeling of the cortico-striatal-thalamo-cerebellar network. **(A)** Intrinsic connections across all models. **(B)** Modulatory connections. For simplicity we show only one hemisphere but the modulatory connections are assumed in both. The upper row describes models within the “basal ganglia pathway” with different modulations of the motor-putamen-thalamo-motor circuit. The bottom row describes models within the “cerebellar pathway” with different modulations of the motor-cerebello-thalamo-motor network. M1, primary motor cortex; Pu, putamen; SMA, supplementary motor area; PMC, premotor cortex; TH, thalamus; CB, cerebellum. The winning model is marked in green.

We used dynamic causal modeling (DCM, Friston et al., 2003) as implemented in SPM12 (v. 7771), version DCM12.5, to investigate changes in effective connectivity within a cortico-striato-thalamo-cerebellar network due to impaired learning and motor performance in PD. Importantly, we hypothesized that connectivity patterns within this network would differ in PD compared to healthy controls. To this end, 12 nodes were specified containing bilateral motor cortical areas: primary motor cortex (M1), supplementary motor area (SMA), and premotor cortex (PMC) as well as putamen, thalamus and cerebellum. These specific nodes were chosen based on our previous work showing that connectivity between these regions is modulated by motor sequence learning in healthy subjects (Tzvi et al., 2014, 2015; Liebrand et al., 2020). For PD, the thalamus and its’ connectivity patterns within this network is of special interest as dopaminergic denervation in SN in PD is known to affect thalamo-cortical projections leading to movement deficits in PD. We therefore included the thalamus as well in the modeled network in this study. Within each hemisphere and the contralateral cerebellum, all VOIs were assumed to be fully connected (Figure 2A). We also allowed an intrinsic connection between homolog M1, based on previous work (Tzvi et al., 2017) showing that a model with a connection between homolog M1 is preferable over models that included other homolog connections. In addition, our previous fMRI studies of MSL showed that specifying the right cerebellum as input node leads to highest exceedance probability (Tzvi et al., 2014, 2015, 2017). We therefore chose this node as input here as well in order to minimize inclusion of less probable model structures. Note that it is likely that the driving input is mediated by un-modeled regions such as the visual cortex. The input and connections specified here do not necessarily represent anatomical input and connectivity but rather a “net effect.”

We then specified 14 different models which allowed modulation of different connections (Figure 2B). These were based on:

(1). “Basal-ganglia pathway”: these models are based on the classical model of direct and indirect pathways in the basal ganglia underlying motor actions. According to this model, cortical activation leads to activation of striatum, which in turn leads to disinhibition of the thalamus which projects to the cortex (Calabresi et al., 2014). The seven models specified here model modulation by the motor task of the entire circuit (model BG pathway 1) or of parts of the circuit (model BG pathway 2–7). In PD, dopaminergic denervation in SN leads to abnormal activation of striatal output nuclei and thus an over-inhibition of thalamic neurons projecting to the motor cortex.
(2). “Cerebellar pathway”: here, we specified a network based on the cerebello-thalamo-cortical pathway connecting sensorimotor and premotor areas to cerebellum, from cerebellum to thalamus and from thalamus back to sensorimotor and premotor areas (Ramnani, 2006). Here as well, we modeled modulation of the entire circuit (model CB pathway 1) or of parts of the circuit (model CB pathway 2–7).

### Dynamic Causal Modeling of the Nigral-Striatal Pathway

In addition to the large bilateral cortico-striato-thalamo-cerebellar model above, we tested the hypothesis that degeneration of dopaminergic neurons in SN leads to abnormal activation of striatal output nuclei and stronger inhibition of the thalamus in pwPD. To this end, we implemented a restricted 3-node model accounting for the right SN activity increase in pwPD found in the regional analysis below (see section “fMRI Results”), as well as right putamen and right thalamus nodes (Figure 5A). We allowed intrinsic connections between all nodes. The right putamen was chosen to be the input node, as it receives sensorimotor information via projections from neocortex (Calabresi et al., 2014). The first model tested the modulation of the connection from SN to the thalamus (Calabresi et al., 2014). The second model tested the modulation of SN to thalamus connection based on the hypothesis above.

### Model Selection

For each model, a one-state bilinear system of differential equations was inverted and together with a biophysically motivated hemodynamic model, an estimated BOLD signal was produced. This modeled BOLD signal was then iteratively fitted to the real data through a gradient ascent on the free-energy bound. We describe the results of the DCM analysis on two levels: on a model level and on a parametric level. First, we selected a “winning” model out of a candidate set of equally plausible models, based on its protected exceeding probability, using Bayesian Model Selection. This was done for each analysis (the 12-nodes cortico-striato-thalamo-cerebellar network as well as the 3-nodes nigral-striatal network) and for each group separately. Second, connectivity parameters within the “winning model” were then analyzed using a 2 × 2 mixed effects ANOVA with factors COND (SEQ, RND) and group (PD, controls) to discover learning-specific changes due to PD. We extracted the modulatory parameters from the winning model by averaging across the three sessions. Note that in some cases, the model did not converge, leading to zero parameter estimates. In order to not bias the results toward zero, these sessions were identified using the spm_dcm_fmri_check.m script and removed from further analyses.

### Time Series Extraction

We specified the following as volumes of interest (VOIs): primary motor cortex (M1), supplementary motor area (SMA), premotor cortex (PMC), putamen (Pu), thalamus (TH), substantia nigra (SN), and cerebellum (CB). Time series were extracted across all experimental blocks and sessions, in order to account for both learning- and non-learning related changes in the BOLD signal. The coordinates of the sphere centers for each VOI (except for SN) were selected based on the local maxima of the group level task vs. baseline contrast (see Table 4). For SN, we selected the sphere center to be the peak of Group x COND interaction (Table 3). Using a singular value decomposition procedure implemented in SPM12, we computed the first eigenvariate across voxels within 4 mm radius from the sphere center for each subject. Time series were then detrended and sharp improbable temporal artifacts were smoothed by an iterative procedure implementing a 6-point cubic-spline interpolation. Finally, we estimated the explained variance of the signals we extracted from each of the VOIs by computing the proportion of the first eigenvariate in the signal. The minimal variance explained (across all subjects and VOIs) was 62% which means that the first eigenvariate explained much of the signal variance in the different VOIs.

**TABLE 4:** Dynamic causal modeling nodes: Task > Baseline.

Region	Coordinates	Sub-region		*t*-val
L M1	−54 −16 32	BA4		7.03
R M1	56 −16 40	BA4		4.65
L SMA	−6 4 56	BA6		9.43
R SMA	0 2 48			9.85
L PMC	−42 −8 60	BA6		7.68
R PMC	48 −4 56	BA6		8.34
L putamen	−24 −2 6			6.87
R putamen	26 −2 6			6.59
L cerebellum	−24 −66 −56	Lobule VIII		4.65
R cerebellum	16 −64 −54	Lobul	Lobule VIII	4.16
L thalamus	−14 −16 8			4.08
R thalamus	14 −14 6			2.39

## Results

### Behavioral Results

#### Reaction Times

We analyzed changes in reaction times (RT) due to learning and PD using a repeated-measures analysis of variance (rmANOVA) with factors Group (PD, controls), Condition (SEQ, RND) and Block (1–6). Generally, pwPD were significantly slower in responding to the stimulus, across both SEQ and RND blocks, compared to healthy controls, as reflected by a main effect of Group [*F*_(1, 30)_ = 5.5, *p* = 0.03, Figure 1B]. No other main effects or interactions were found (*p* > 0.1). We also found no effect of gender on learning when using rmANOVA with factors group (male, female), Condition (SEQ, RND) and Block (1–6) (all main effects or interactions: *p* > 0.3). We then investigated whether subjects of both groups learned the underlying motor sequence using two separate rmANOVA with factors Condition and Block in each group. Neither PD nor controls showed specific sequence learning effects reflected by either a main effect of Condition (both *p* > 0.1) or a significant Condition × Block interaction (both *p* > 0.2).

However, a main effect of Block in the control group [*F*_(5, 75)_ = 2.8; *p* = 0.02] suggested general task improvement independent of the condition (Figure 1C), which was not evident in PD (*p* = 0.8, Figure 1D). We then used a two-sample *t*-test to explore whether task improvement differed between PD and controls. First, RT were normalized by the averaged RT across the task in each subject (Figures 1C,D). Changes in normalized RT across the task thus represent general learning effects, irrespective of individual RT performance. Then, we specified a Performance Index using the difference between normalized RTs averaged across the first three blocks of the task (one random and two sequence blocks with 24 trials each) and normalized RT averaged across the three last blocks of the task (see gray squares in Figures 1C,D). Indeed, we found that the Performance Index was larger in controls compared to PD (*t*_30_ = 2.1, *p* = 0.04), suggesting the pwPD had deficits in learning stimulus-response associations in this task. This effect was not related to motor deficits in PD, evaluated using the UPDRS III score (*p* > 0.8) but did tend to relate to levodopa equivalent dose (LED) such that larger LED was associated with worse performance at the end of the task (Figure 1E, *r* = −0.48, *p* = 0.057). There was no correlation between the Performance Index and disease duration (*p* > 0.2).

#### Error Rates

In terms of error-rates, pwPD produced significantly more errors compared to healthy controls (*Z* = 2.75, *p* = 0.006; Figure 1F). Similar to the RT analysis above, condition differences in error-rates were assessed in each group separately. PwPD produced significantly more errors (corrected for multiple comparisons) in RND compared to SEQ in block 1 (*Z* = 2.59, *p* < 0.01), block 2 (*Z* = 2.36, *p* = 0.02), and block 5 (*Z* = 2.71, *p* = 0.007). In healthy controls, error rates also tended to be larger in RND compared to SEQ in block 2 (*Z* = 2.30, *p* = 0.02), block 3 (*Z* = 1.96, *p* < 0.05), and block 6 (*Z* = 2.59, *p* < 0.01) but this difference did not survive correction for multiple comparisons. These differences indirectly suggest that some learning of the sequence has taken place in both groups. Further, we explored whether condition difference in error-rates were associated with motor deficits in PD, evaluated using the UPDRS III score, LED and disease duration. No relationship was found with UPDRS III (*p* > 0.7), disease duration (*p* > 0.7), and levodopa equivalent dose (LED) (*p* > 0.3).

In sum, while RT analysis showed no condition differences in both healthy controls and pwPD, the significantly higher error-rates during RND blocks indicate that some learning of the sequence has taken place. Healthy controls improved condition unspecific task performance as indicated by faster RTs at the end of the task compared to the beginning whereas pwPD could not. This suggests that pwPD are impaired in learning stimulus-response associations.

### Functional MRI Results

#### Decrease in Left Hippocampus Activity Associated With General Task-Related Deficits in PD

**FIGURE 3:**
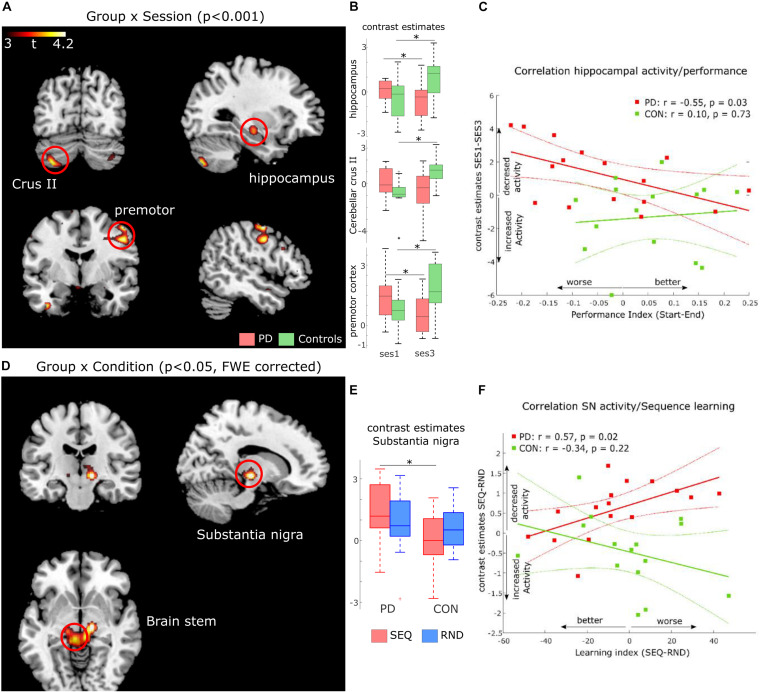
FMRI results. **(A)** Activation maps for Group (PD, controls) x Session (SES1, SES3) interactions at p < 0.001, uncorrected voxel-level threshold. **(B)** Contrast estimates derived from activation clusters shown in A. Boxplots show significant increased activity in controls and decreased activity in PD. **(C)** Correlation between activity increase in hippocampus and improved performance in both PD (red, significant) and healthy controls (green, non-significant). **(D)** activation maps for Group (PD, controls) × Condition (Sequence, Random) interactions at *p* < 0.05, cluster-level family-wise error corrected. **(E)** Contrast estimates derived from the activation cluster in substantia nigra (SN) shown in **(D)**. Boxplots show significant increased activity in PD compared to controls during sequence blocks. **(F)** Correlation between activity increase in SN and worse sequence learning in both PD (red, significant) and healthy controls (green, non-significant).

To investigate changes in general task-related activity due to PD, we subjected the contrasts of Session 1 and Session 3 in each group to a flexible factorial design with factors Group (PD, controls) and Session (SES1, SES3). There were no significant clusters for the Group factor. Although no significant Group × Session interactions were observed on a family-wise error (FWE) corrected p-level, a few large clusters in right premotor cortex, left insula, left cerebellum crus II, and left hippocampus were evident on an uncorrected *p* < 0.001 voxel-based significance level (see Table 3 and Figure 3A). Group x Session interactions show regions associated with both task-related changes over time as well as differences between PD and controls (Figure 3B). Post-hoc paired *t*-tests show that these interactions arise from a specific increase from SES1 to SES3 in healthy controls (Figure 3B, left cerebellar crus II: *t*_14_ = 3.9, *p* = 0.002; right premotor cortex: *t*_29_ = 3.4, *p* = 0.004; left hippocampus: *t*_29_ = 2.2, *p* = 0.04), with the opposite pattern in PD (Figure 3B, right premotor cortex: *t*_15_ = 2.5, *p* = 0.02, left hippocampus: *t*_15_ = 2.6, *p* = 0.02, left cerebellar crus II: ns). In addition, two-sample *t*-tests comparing both groups revealed decreased activity in PD compared to controls in SES3 (Figure 3B, left cerebellar crus II: *t*_29_ = 2.8, *p* = 0.008; right premotor cortex: *t*_29_ = 3.1, *p* = 0.004; left hippocampus: *t*_29_ = 2.9, *p* = 0.007). These results suggest that healthy controls recruit regions important for task performance, whereas in pwPD, decreased activity in these regions could be related to deficits in general task performance.

To test this hypothesis, we explored two relationships in the PD group. First, we investigated a link between activity changes activity from SES1 to SES3 and the Performance Index (see Table 2). Second, we explored a link between activity changes activity from SES1 to SES3 and measures of disease progression, namely LED and disease duration. We found a negative correlation between activity changes in left hippocampus and the Performance Index (Figure 3C, *r* = −0.55, *p* = 0.03), which meant that pwPD who slowed toward the end of the task, i.e., performed worse, also showed relatively decreased left hippocampus activity in SES3 compared to SES1. Second, we found a positive correlation between activity changes in left cerebellar crus II and LED (*r* = 0.53, *p* = 0.03, Supplementary Figure 1A) as well as disease duration (*r* = 0.59, *p* = 0.02, Supplementary Figure 1B). This suggests that increased dopaminergic denervation, reflected in disease duration and higher daily doses of dopaminergic medication, is associated with decreased left cerebellar crus II activity from SES1 to SES3. Together, these results suggest that impaired learning of stimulus-response associations in PD is associated with a hippocampal decrease in activity, whereas the severity of the disease affects recruitment of left cerebellar crus II.

#### Increased SN Activity Associated With Sequence Learning Deficits in PD

Based on the behavioral results (error-rates) that indirectly suggest sequence-specific learning in both groups, we assessed possible differences in neural responses to implicit MSL in PD compared to controls, using a flexible factorial design with factors Group and Condition (SEQ vs. RND across all sessions). We found a Group × Condition interaction in a large cluster with peak activity in left substantia nigra (SN, *t*_59_ = 4.47, *p* < 0.05, FWE cluster-level corrected). The cluster included adjacent bilateral midbrain areas (Table 3 and Figure 3D). Note that this activity was found on a whole-brain analysis despite imaging parameters not optimized to locate activity in SN. Post-hoc paired *t*-tests show that this interaction was caused by decreased activity in healthy controls compared to relatively increased activity in PD during SEQ blocks (Figure 3E, *t*_29_ = 2.7, *p* = 0.01). Differences in RND were non-significant (*p* = 0.5).

To determine whether these differences in neural activity were related to individual sequence learning, we first created a Learning Index based on RT differences between SEQ and RND averaged across SES2 and SES3 (blocks 3–6). Note that this Learning Index is different than the Performance Index specified above (Table 2). We then correlated the Learning Index with learning-related activity (SEQ-RND) in SN. We found a significant positive correlation (*r* = 0.57, *p* = 0.02; Figure 3F) in the PD group, which meant that stronger activity in sequence blocks was driven by reduced learning. In addition, learning-related (SEQ-RND) activity in SN tended to positively correlate with disease duration (*r* = 0.51, *p* = 0.05, Supplementary Figure 2B) as well as levodopa equivalent dose (*r* = 0.47, *p* = 0.06, Supplementary Figure 2A). This suggests that the severity of the disease, reflected in its duration and higher dopaminergic medication, lead to increased activation of SN during learning of a motor sequence. There was no association between learning-related (SEQ-RND) activity in SN and the UPDRS score (*p* > 0.1).

### Dynamic Causal Modeling of the Cortico-Striato-Thalamo-Cerebellar Network

#### Condition Differences in Modulation of Motor and Premotor Areas to Cerebellum

Using Dynamic Causal Modeling (DCM), we next asked whether striatal dysfunction in pwPD leads to changes in connectivity in a cortico-striato- cerebellar network underlying motor learning. To this end, we specified two pathways, along which modulation by learning and by task performance may be evident. First, a “basal ganglia circuit,” connecting motor and premotor cortex to putamen, then to thalamus and back to motor cortical regions; second, a “cerebellar circuit” connecting motor and premotor cortex to cerebellum, then to thalamus and back to motor cortical regions (see Figure 2B). Further details regarding the two circuits can be found in section 2.7 of the methods. In one pwPD and one healthy control, the model did not converge in any session, leading to exclusion of these two subjects for the DCM analyses only.

**FIGURE 4:**
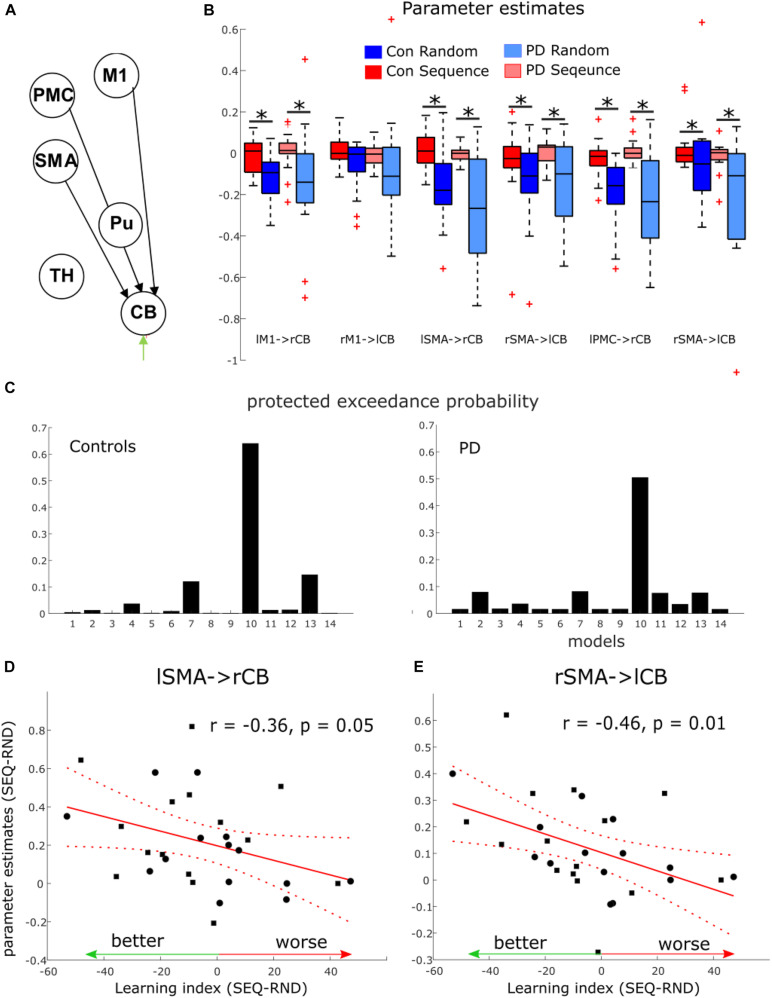
Dynamic causal modeling results of the cortico-striatal-thalamo-cerebellar network. **(A)** Winning model in both groups. Note that the model had 12 nodes in both hemispheres. Here only 6 nodes in left cerebrum and right cerebellum are depicted. M1, primary motor cortex; PMC, premotor cortex; SMA, supplementary motor area; Pu, putamen; TH, thalamus; CB, cerebellum. **(B)** Boxplots for parameter estimates in the winning model (A) for both groups (PD, controls) and conditions (Sequence, Random). Significant differences are marked with a star. **(C)** Protected exceedance probability in healthy controls and PD showing model 10 (CB pathway 3) was favorable over the other models (Figure 2). **(D,E)** Correlation between condition differences in modulation of SMA–> CB connection and learning index.

A total of 14 models were compared using Bayesian Model Selection, 7 models for each pathway (Figure 2B). Random-effects Bayesian Model Selection showed that CB pathway 3 (Figure 4A) had the strongest protected exceedance probability (Figure 4C) in both PD (best model: 0.50, next model: 0.08) and controls (best model: 0.65, next model: 0.15). Comparison between models are shown in Figure 4C. In this model, shown in Figure 4A, connections from bilateral M1, premotor cortex (PMC) and supplementary motor area (SMA) to contralateral cerebellum (CB) were modulated by the motor task (both SEQ and RND blocks). Next, we tested differences in modulatory parameters between PD and controls in the six connections of the winning model (bilateral M1, SMA and PMC–> CB), using a 2 × 2 mixed effects ANOVA with factors COND (SEQ, RND) and group (PD, controls). We found in all connections (except for right M1–> left CB), a significantly larger negative modulation in RND compared to SEQ (Figure 4B and Table 5). There were no differences between PD and controls in terms of modulatory parameters (*p* > 0.1 for the group factor for all modulatory connections).

**FIGURE 5:**
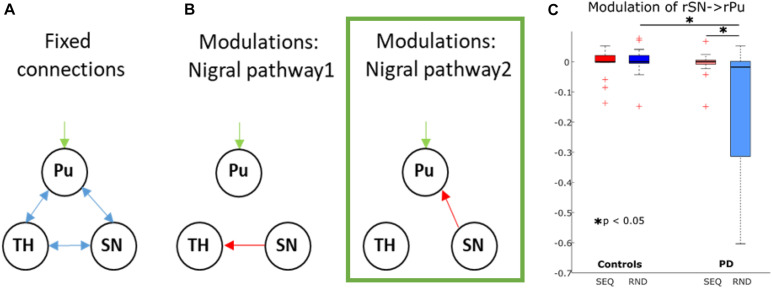
Dynamic causal modeling of the nigro-striatal pathway. **(A)** Intrinsic connections across all models. **(B)** Modulatory connections. Pu, putamen; TH, thalamus; SN, substantia nigra. The winning model is marked in green. **(C)** Boxplots for parameter estimates in the winning model (B) for both groups (PD, controls) and conditions (Sequence, Random). Significant differences are marked with a star.

**TABLE 5:** Modulatory parameters in the winning cortico-striato-thalamo-cerebellar model (main effect of COND: RND > SEQ).

	*F*-val	*p*-val
lM1–> rCB	7.2	0.013
rM1–> lCB	0.9	0.357
lSMA–> rCB	23.0	<0.001
rSMA–> lCB	13.2	0.001
lPMC–> rCB	23.3	<0.001
rPMC–> lCB	10.8	0.003

#### Modulation of SMA to Cerebellar Connections Associated With Better Learning

We then asked whether condition differences in modulatory effects on connections from M1, SMA and PMC to CB reflect implicit learning of the underlying sequence. As no group differences were evident, we correlated the Learning Index (see Table 2) across all subjects of both groups, with the individual condition differences in modulatory parameters. We found a significant negative correlation in a connection from right SMA to left cerebellum (Figure 4E, *r* = −0.46, *p* = 0.01) and a tendency for a negative correlation in a connection from left SMA to right cerebellum (Figure 4D, *r* = −0.36, *p* = 0.05). These negative correlations suggest that the stronger the difference in modulation between SEQ and RND, the better the subject learnt.

In addition, we tested the hypothesis that endogenous connections from putamen (Pu) to thalamus (TH) are altered in PD compared to controls. There were no differences in left Pu–> left TH connection (controls 0.04 ± 0.03; PD: 0.02 ± 0.01, *p* = 0.4), as well as in right Pu–> right TH connection (controls 0.02 ± 0.02; PD: 0.05 ± 0.01, *p* = 0.3). In terms of the driving input to bilateral cerebellum by the task, we found a significant increase in the effect of task input during RND compared to SEQ blocks [right cerebellum: *F*_(1, 27)_ = 24.7, *p* < 0.001; left cerebellum: *F*_(1, 27)_ = 16.3, *p* < 0.001]. There were no differences between the groups (*p* > 0.1).

### Dynamic Causal Modeling of the Nigro-Striatal Pathway

While no SN activity-induced effect was observed on the broad cortico-striato-thalamo-cerebellar network, we additionally explored, in a more compact right-lateralized model, whether the increased SN activity during sequence-specific learning in pwPD affected activity in putamen and thalamus, as predicted by basal ganglia models of motor control (Calabresi et al., 2014). Two models were compared using Bayesian Model Selection: the first allowing modulation of SN to thalamus and the second allowing modulation of SN to putamen (Figure 5A). In all subjects, both models converged at least in one session, meaning that no subjects were excluded from this analysis. Random-effects Bayesian Model Selection showed that model 2 (Figure 5B) had the strongest protected exceedance probability in both PD (0.97) and controls (0.61). Using a 2 × 2 mixed effects ANOVA with factors COND (SEQ, RND) and group (PD, controls), we tested for differences in modulatory effects between PD and controls. We found a main effect of group [*F*_(1, 29)_ = 5.7, *p* = 0.02], a main effect of COND [F_(1, 29)_ = 4.3, *p* = 0.047], and COND × group interaction [F_(1, 29)_ = 5.1, *p* = 0.03]. *Post-hoc* tests showed a stronger negative modulation of SN to putamen connectivity during RND in pwPD compared to controls (*t*_29_ = 2.3, *p* = 0.03, Figure 5C), with no such differences for SEQ (*p* > 0.9). In addition, negative modulation of SN to putamen connectivity was significantly stronger in RND compared to SEQ in pwPD (*t*_15_ = 2.3, *p* = 0.03, Figure 5C). No condition differences were observed in controls (*p* > 0.7). Endogenous connections between all nodes were positive and did not differ between the groups (all *p* > 0.2). There were no correlations between modulation of SN to putamen connectivity and the Learning Index or the levodopa equivalent dose.

## Discussion

The aim of this study was to investigate the neural correlates underlying implicit motor sequence learning (MSL) deficits in PD. We found that neither healthy controls nor pwPD demonstrated significant sequence-specific learning across blocks of practice. Although online MSL has been shown to be relatively preserved depending on task complexity in healthy elderly subjects (see King et al., 2013), this suggests that implicitly learning a 12-item finger movement sequence is too difficult a task in this elder age-group. However, subjects of both groups made consistently more errors during random (RND) blocks compared to sequence (SEQ) blocks, suggesting that at least some chunks of the sequence were encoded. In addition, we found general task performance (stimulus-response association) deficits in PD, which could be related to nigral neurodegeneration and resulting reduction of dopaminergic neurotransmission.

Analysis of fMRI data showed decreased activity in pwPD across blocks of task performance in hippocampus as well as right premotor cortex. These results suggest a link between task-related deficits and recruitment of these regions. Indeed, we found that activity decrease over time in left hippocampus was associated with worse task performance over time in the PD group, highlighting a possible role of hippocampus in deficient learning of stimulus-response associations in PD. Importantly, activation of the substantia nigra (SN) during sequence learning was evident in the PD group only, and the magnitude of this activation was inversely correlated with learning. In addition, this increased activity in SN tended to correlate with LED and disease duration. One way to interpret this finding is that disease progression led to “over-recruitment” of the remaining viable dopaminergic neurons in SN during sequence learning. However, whether degeneration of nigral dopaminergic neurons or over-recruitment of viable SN neurons is associated with learning deficits remains open.

Finally, we investigated effective connectivity in the cortico-striato-thalamo-cerebellar network as well as in a restricted nigral-striatal network. There were no differences between pwPD and controls were evident in the cortico-striato-thalamo-cerebellar network, suggesting that both pwPD and controls modulate the same cortico-cerebellar circuit during task performance. However, modeling the nigro-striatal network revealed that connection from SN to putamen was more negatively modulated in pwPD compared to controls in RND (but not in SEQ). We speculate that connectivity from SN to putamen in pwPD is equivalent to healthy controls when dopaminergic neurons in SN are recruited for sequence learning, but returns to a pathological state when no learning occurs.

### Evidence for Implicit Motor Sequence Learning in PD and Healthy Controls

In general, reaction times (RT) in pwPD were significantly slower compared to healthy controls. General slowness probably reflects both bradykinesia and cognitive deficits in executive functions in PD (Cooper et al., 1994; Leis et al., 2005). On the group level, neither pwPD nor age-matched healthy controls improved RT during performance of the implicit 12-element sequence, when compared to random trials with no underlying sequential pattern. However, subjects of both groups made significantly more errors during RND compared to SEQ blocks. This effect is consistent with our previous findings in MSL tasks in healthy young subjects, in which larger error-rates in RND compared to SEQ blocks were found (Tzvi et al., 2015, 2016; Liebrand et al., 2020). Previously, we interpreted this effect as an implicit attempt to perform the sequence or chunks of the sequence in RND trials following sequence learning, however, unsuccessfully. Thus, these results indicate that despite a lack of improvement in RT, some implicit sequence-specific learning was evident in all subjects. Note that using this exact same task, we have previously shown that younger healthy controls were faster during SEQ compared to RND blocks, while persons with cerebellar degeneration were not (Tzvi et al., 2017). Together with the current results, we suggest that age and neurological deficits may impact the ability to improve RT in this task. The absence of sequence learning differences between pwPD and their healthy controls is surprising as common findings usually point to task-specific learning deficits in PD, using various sequence lengths, and mixtures of SEQ and RND blocks (for a systematic review see Ruitenberg et al., 2015). However, not all studies report the error-rates, which are an important indicator for learning. For example, using a 12-element sequence of button presses and a mix of RND and SEQ blocks, Seidler et al. (2007) found that pwPD did not speed up performance in SEQ compared to RND blocks, while healthy controls did. Importantly, the authors found increased error-rates in RND compared to SEQ blocks in both groups, in line with the findings reported here.

In terms of general task performance (i.e., independent from condition), healthy controls showed significantly improved RT at the end of the task compared to the beginning, i.e., a better Performance Index (Table 2), while pwPD did not improve RT across blocks of task execution. In addition, a worse Performance Index in PD correlated (on trend level, *p* = 0.05) with their daily LED. As higher LED may be indicative of more advanced disease stages, this suggests that the ability to learn simple stimulus-response associations is affected by disease progression. Notably, this effect could not be explained by PD-associated reduced motor abilities (i.e., reduced movement speed due to bradykinesia), as the UPDRS-III score showed no correlation with the Performance Index.

### Decreased Hippocampal Activity Is Associated With Deficient Stimulus-Response Associations in PD

To obtain mechanistic insights into the possible stimulus-response deficits in PD, we analyzed differences in task-related changes of neural activity over time, between pwPD and healthy controls. We found large clusters in left cerebellum crus II, right premotor cortex, left hippocampus and left insula, along with other smaller clusters. Specifically, these regions showed increased activity in healthy controls, and decreased activity in PD, suggesting that deficient learning of stimulus-response associations in PD is related to decreased activity in these regions. Indeed, we found that activity decrease over time in left hippocampus was associated with worse task performance over time in the PD group. The hippocampus is well-known for its important role in spatial memory encoding (Burgess et al., 2002), as well as in rapid acquisition of visuomotor mappings (Wise and Murray, 1999). Progressive hippocampal atrophy has been previously observed in pwPD (Camicioli et al., 2003; Brück et al., 2004) and was related to impaired memory (Jokinen et al., 2009). Calabresi et al. (2013) suggest that imbalanced interaction between dopamine transmission and hippocampal synaptic plasticity might play a role in learning and memory deficits observed in PD. Thus, our results suggest that deficient ability to learn stimulus-response mappings is associated with hippocampal dysfunction in pwPD. Note, however, that these results should be treated with caution as these effects were found on an uncorrected p-level threshold of *p* = 0.001. Future studies with larger cohorts may try to replicate these results by specifically investigating changes in activity patterns during deficient stimulus-response mapping in PD. Interestingly, task-related decrease in activity of left cerebellar crus II was significantly correlated with indirect markers of disease progression (LED, disease duration). This may indicate that intact endogenous nigro-striatal dopaminergic stimulation is necessary to adequately recruit cerebellar crus II across repeated task execution. This effect may be related to well described associations of pathological changes in the cerebellum in PD with dopaminergic degeneration, abnormal drives from the subthalamic nucleus, and dopaminergic treatment (see review by Wu and Hallett, 2013).

### Increased Substantia Nigra Activity Is Predictive of Motor Sequence Learning Impairment in PD

Investigation of the underlying differences between pwPD and healthy controls in neural responses to MSL revealed sequence-specific activity difference comparing pwPD to controls, in a cluster with a peak in the right substantia nigra (SN) and adjacent bilateral midbrain areas. While healthy controls showed no learning-specific increase of SN activity, in pwPD, SN activity significantly increased. Correlation analysis in this cluster showed that stronger activity in SN was associated with worse sequence-specific learning in the PD group. While increased SN activity tended to correlate with LED and disease duration, no relationship between the Learning Index (Table 2) or the UPDRS-III score with LED was found. Daily dosages of dopaminergic medication as well as disease duration likely indicate the state of dopaminergic denervation in SN and thus the dysfunction of endogenous nigro-striatal dopaminergic stimulation. Previous studies show that lesions to SN in a rat model of PD lead to loss of striatal dopamine and deficits linked to habit learning and working memory (Da Cunha et al., 2002). In addition, recordings of SN neurons in pwPD performing a memory task showed that dopaminergic neurons in SN are modulated by memory (Kamiński et al., 2018). Matsumoto and colleagues (Matsumoto et al., 1999) showed that depletion of nigrostriatal dopamine in primates leads to a specific deficit in learning new motor sequences. Interestingly, while exogenous dopamine replacement therapy could restore high-speed walking after genetic ablation of dopaminergic neurons in the SN of mice, motor skill learning remained severely impaired (Wu et al., 2019). This suggests that the process of acquiring new motor skills relies on endogenous nigro-striatal dopamine release that cannot be compensated by exogenous dopamine replacement therapy (although cardinal motor symptoms of PD such as bradykinesia are effectively attenuated).

In sum, the above body of evidence indicates that impairments in the capacity to learn sequential movements in PD is related to increasing demands on the dysfunctional endogenous dopaminergic system. Therefore, the correlation between LED/disease duration and activity increase in SN during sequence performance may point to an increased (yet insufficient) drive to recruit the dysfunctional endogenous nigro-striatal dopaminergic system that is essential for MSL. As all pwPD were on their regular dopaminergic medication during the experiment, it seems improbable that increased SN activity and the associated impairment in MSL were a consequence of insufficient global dopamine availability during learning. Our finding rather supports the notion that MSL relies on sequence learning-specific recruitment of the endogenous nigro-striatal dopaminergic system. In the healthy brain, SN activity has not been associated with motor sequence learning (Meta analysis by Hardwick et al., 2013), perhaps since the functional nigro-striatal system leads to learning-related changes in striatal output nuclei instead, which explains the common finding of putamen, caudate as well as thalamic activity changes with motor sequence learning in healthy subjects (Hardwick et al., 2013).

Interestingly, decreased activity in the hippocampus during task performance and learning-specific increased activity in SN found here in pwPD could be linked together. The hippocampus receives dopaminergic input from SN and thus could influence learning and memory processes in PD due to decrease in dopamine availability resulting from SN denervation (Lisman and Grace, 2005). Studies suggest that dopaminergic neurons in SN encode stimulus novelty which serve declarative hippocampal memory processes (Bunzeck and Düzel, 2006; Kamiński et al., 2018). Notably the effect of dopaminergic neurons in SN on non-declarative memory process such as in this implicit motor sequence learning task has not been investigated thus far. A plausible scenario is that dysfunctional endogenous nigro-striatal dopaminergic system not only directly affects sequence learning but also indirectly affects stimulus-response associations through an SN-hippocampal loop. This prediction should be tested in future studies.

### Differences in Learning-Specific Modulation of SN-Putamen Connectivity Underlying PD

We examined potential connectivity differences within the cortico-striato-thalamo-cerebellar network which might explain MSL deficits in PD. Specifically, we asked whether motor task performance modulated connections in the “basal-ganglia pathway,” in which the motor command propagates from motor and premotor areas to putamen, thalamus and back to cortical motor areas (Calabresi et al., 2014), or rather in the “cerebellar pathway,” in which motor commands are propagated from motor and premotor areas to cerebellum, and back through the thalamus (Ramnani, 2006). We expected to find evidence for abnormal striatal activity in PD reflected in connections with putamen. Results show that the optimal model in both pwPD and controls had connections from M1 and premotor areas to cerebellum that were modulated by the motor task, replicating our previous findings (Tzvi et al., 2014, 2015, 2017; Liebrand et al., 2020). Importantly, we found no evidence for differences in modulatory parameters between PD and controls, suggesting that these interactions are not affected by abnormal striatal activity in PD.

We therefore conducted an additional analysis exploring whether learning-specific activity increase in SN, shown in the regional analysis above, differentially affected putamen and thalamus in pwPD compared to controls. Indeed, we found that negative modulation of a connection from SN to putamen was significantly larger during RND compared to SEQ in pwPD, and when compared to RND in controls. Similarly, a recent resting-state functional connectivity study showed that pwPD with greater dopaminergic deficits had decreased connectivity between midbrain and putamen (Li et al., 2020). Thus, we speculate that during implicit learning of a motor sequence, the increased drive to recruit the dysfunctional endogenous nigro-striatal dopaminergic system is reflected by similar modulation of SN to putamen connectivity as in controls. However, when no learning pattern is available (RND), the nigro-striatal system returns to its pathological state of decreased connectivity. Note that there was no resting-state condition in our study in order to test this speculation. Future studies may examine causal SN- > putamen interactions underlying both motor performance and rest.

### Implicit Motor Sequence Learning Modulates Cortico-Cerebellar Connections Independent From PD

Exploring connectivity patterns in the winning model of the cortico-striato-thalamo-cerebellar network, we found condition differences in both healthy controls and pwPD, in modulation of bilateral connections from M1, premotor cortex and SMA to cerebellum. Specifically, during RND negative modulation of these connections was evident, whereas modulation by SEQ was close to zero. These results are in accordance with our previous findings in persons with cerebellar degeneration (Tzvi et al., 2017) but in contrast to our findings in healthy young controls (Tzvi et al., 2014), in which negative modulation of this connection was observed during SEQ. Together with the lack of RT differences between SEQ and RND blocks, these results suggest that previously found negative modulation in SEQ relate to improved RT, whereas here, negative modulation during RND could relate to higher error-rates. Importantly, we observed that modulation of SMA to cerebellum connection was associated with the Learning Index (Table 2), such that subjects who were better in sequence learning, also had stronger condition differences in modulation of this connection, probably driven by larger negative modulation during RND. Given the important role of SMA in forming sequential representations of movements (Cona and Semenza, 2017), it is conceivable that subjects who better encoded the underlying sequence also implicitly expected sequential patterns to appear during RND. The lack of sequential pattern then led to decreased communication from SMA to cerebellum.

### Limitations

Some limitations of this study need to be mentioned. First, the lack of RT differences between SEQ and RND in both pwPD and healthy controls suggest that on the group level no learning has taken place. Note, however, that error-rate differences between conditions can also be indicative of learning, albeit indirectly. Second, although 23 pwPD were originally included in the study, for various reasons, the data of only 16 pwPD could be analyzed. This relatively small sample size may have led to the marginal differences between pwPD and controls in terms of neural activity underlying general task improvements, as well as the trending but non-significant relationships with parameters of disease progression (LED, disease duration). Note however, that the main finding of the study, namely learning-specific activity increase in SN of pwPD was detected on a whole brain voxel level threshold of *p* = 0.001 (corrected across the cluster with family-wise error threshold of *p* = 0.05), suggesting that the effect was strong enough to be detected also by a relatively small group of subjects. Third, we examined several relationships between behavioral data as well as activation changes and parameters of disease progressions. As these tests were exploratory, we did not perform any corrections for multiple comparisons. Future studies should scrutinize the relationship between behavioral measures of MSL, changes in activity and parameters of disease progressions. Finally, it would have been of great interest to explore the differential contribution of SN pars compacta (SNc), known to project to putamen, and SN pars reticulata (SNr) which projects to the thalamus, on the nigral-striatal pathway. However, the limited spatial resolution does not allow to differentiate these two SN sub-structures. Future studies could focus only the basal ganglia using high-resolution functional MRI in order to specifically investigate the contribution of each sub-structure and its connectivity with putamen and thalamus to motor learning.

## Conclusion

In this study we investigated the neural correlates underlying implicit MSL deficits in PD. Increase in error-rates during RND compared to SEQ in both groups suggested that some sequence learning was evident. FMRI analysis revealed two noteworthy findings: first, pwPD showed decreased hippocampal activity with time, and this decrease was associated with worse task performance. Second, pwPD exhibited abnormal activity increase in SN during SEQ blocks specifically, which was associated with worse learning of the underlying sequence. This sequence-specific increase in SN activity in pwPD may point to an increased drive to recruit the dysfunctional endogenous nigro-striatal dopaminergic system for the purpose of learning. Finally, we found no group differences in connectivity within a cortico-striato-thalamo-cerebellar network but a relative decrease in SN- > putamen connectivity in pwPD (compared to controls) specifically in non-learning periods. We speculate that learning induces an increase in SN- > putamen interaction, which returns to a pathological state during no-learning periods.

## Data Availability Statement

The raw data supporting the conclusions of this article will be made available by the authors, upon reasonable request.

## Ethics Statement

The studies involving human participants were reviewed and approved by the Ethics Committee of the University of Lübeck. The patients/participants provided their written informed consent to participate in this study.

## Author Contributions

ET, UK, TM, and MN contributed to the conception and design of the study. ET and RB collected the data. MN and NB performed the medical examination of the PD patients. ET analyzed the data. ET and J-JR wrote the manuscript. All authors contributed to manuscript revision, read, and approved the submitted version.

## Conflict of Interest

The authors declare that the research was conducted in the absence of any commercial or financial relationships that could be construed as a potential conflict of interest.

## Footnotes

1 www.neurobs.com

2 http://www.fil.ion.ucl.ac.uk/spm/

## Abbreviations

DCM: dynamic causal modeling
fMRI: functional magnetic resonance imaging
LED: levodopa equivalent dose
MSL: motor sequence learning
pwPD: persons with Parkinson’s disease
RT: reaction times
RND: random
SEQ, sequence. SN: substantia nigra.
