# Supplementary material for: Motor Sequence Learning Deficits in Idiopathic Parkinson’s Disease Are Associated With Increased Substantia Nigra Activity
Source: Front Aging Neurosci. 2021 Jun 14;13:685168. doi: 10.3389/fnagi.2021.685168 (PMC8236713; doi:10.3389/fnagi.2021.685168)
Supplement: Supplementary file 1 [file Table_1.DOCX]

# Supplementary materials

**Motor sequence learning deficits in idiopathic Parkinson’s disease are associated with increased substantia nigra activity**

Elinor Tzvi^1^, Richard Bey^2^, Matthias Nitschke^2^, Norbert Brüggemann^1,4^, Joseph Classen^1^, Thomas F. Münte^2,3,5^, Ulrike M. Krämer^2,3,5^, Jost-Julian Rumpf^1^

^1^ Department of Neurology, University of Leipzig, Liebigstraße 20, 04103 Leipzig, Germany

^2^ Department of Neurology, University of Lübeck, Ratzeburger Allee 160, 23562 Lübeck, Germany

^3^ Department of Psychology, University of Lübeck, Ratzeburger Allee 160, 23562 Lübeck, Germany

^4^ Institute of Neurogenetics, University of Lübeck, Ratzeburger Allee 160, 23562 Lübeck, Germany

^5^ Center for Brain, Behavior and Metabolism, University of Lübeck, Ratzeburger Allee 160, 23562 Lübeck, Germany


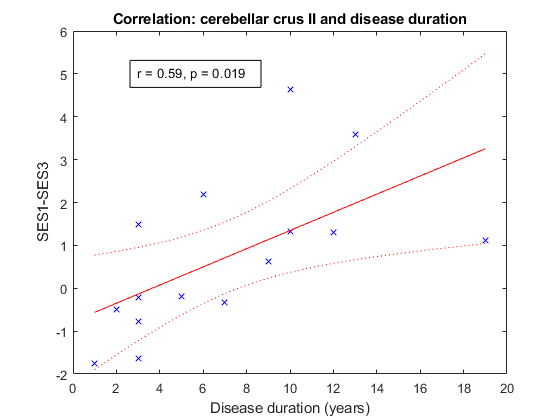

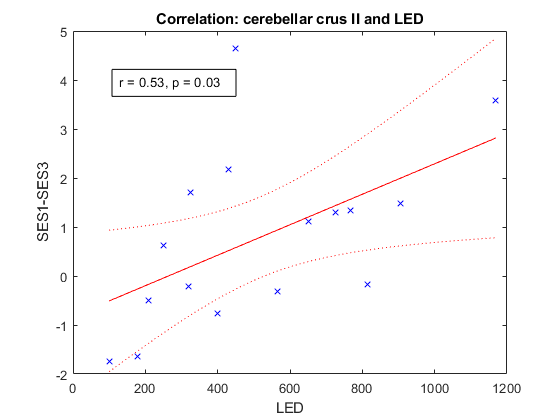


B

A

Figure 1. Significant positive correlations between activity changes in cerebellar crus II from SES1 to SES3 and levodopa equivalent dose (LED) in A and disease duration in B.


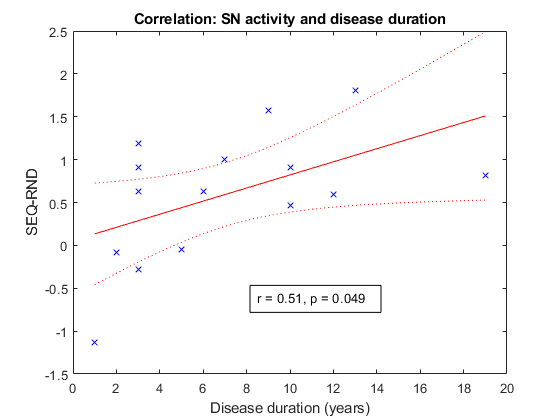

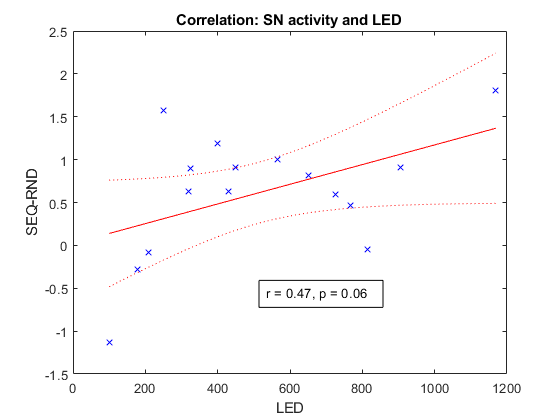


B

A

Figure 2. Trending positive correlations between learning-specific (sequence –random) activity changes in substantia nigra and levodopa equivalent dose (LED) in A and disease duration in B.
